# Endothelial cells adopt a pro-reparative immune responsive signature during cardiac injury

**DOI:** 10.26508/lsa.202201870

**Published:** 2023-11-27

**Authors:** Hali Long, Jeffrey D Steimle, Francisco Jose Grisanti Canozo, Jong Hwan Kim, Xiao Li, Yuka Morikawa, Minjun Park, Diwakar Turaga, Iki Adachi, Joshua D Wythe, Md Abul Hassan Samee, James F Martin

**Affiliations:** 1 Interdepartmental Program in Integrative Molecular and Biomedical Sciences, Baylor College of Medicine , Houston, TX, USA; 2 Department of Integrative Physiology, Baylor College of Medicine , Houston, TX, USA; 3 Cardiomyocyte Renewal Laboratory, The Texas Heart Institute , Houston, TX, USA; 4 Section of Critical Care Medicine, Department of Pediatrics, Baylor College of Medicine , Houston, TX, USA; 5 Section of Cardiothoracic Surgery, Department of Surgery, Baylor College of Medicine , Houston, TX, USA; 6 Cardiovascular Research Institute, Baylor College of Medicine , Houston, TX, USA; 7 Center for Organ Repair and Renewal, Baylor College of Medicine , Houston, TX, USA

## Abstract

Modulation of the heart’s immune microenvironment is crucial for recovery after ischemic events such as myocardial infarction (MI). Endothelial cells (ECs) can have immune regulatory functions; however, interactions between ECs and the immune environment in the heart after MI remain poorly understood. We identified an EC-specific IFN responsive and immune regulatory gene signature in adult and pediatric heart failure (HF) tissues. Single-cell transcriptomic analysis of murine hearts subjected to MI uncovered an EC population (IFN-ECs) with immunologic gene signatures similar to those in human HF. IFN-ECs were enriched in regenerative-stage mouse hearts and expressed genes encoding immune responsive transcription factors (*Irf7*, *Batf2*, and *Stat1*). Single-cell chromatin accessibility studies revealed an enrichment of these TF motifs at IFN-EC signature genes. Expression of immune regulatory ligand genes by IFN-ECs suggests bidirectional signaling between IFN-ECs and macrophages in regenerative-stage hearts. Our data suggest that ECs may adopt immune regulatory signatures after cardiac injury to accompany the reparative response. The presence of these signatures in human HF and murine MI models suggests a potential role for EC-mediated immune regulation in responding to stress induced by acute injury in MI and chronic adverse remodeling in HF.

## Abstract (precis)

scRNA-seq uncovers an endothelial cell (EC) subpopulation with immune responsive signatures in cardiac injury. Profiling of human heart failure samples reveals EC-specific immune responsive signatures in adult and pediatric heart failure. ECs can adopt immune responsive signatures during cardiac stress.

## Introduction

Heart disease, the leading cause of mortality in the world, results from irreversible cardiomyocyte loss after tissue damage such as ischemic injury (Roth et al, 2020; Virani et al, 2021). Extensive basic, translational, and clinical research efforts have been dedicated to developing therapies to combat heart disease. To date, however, no available therapy can permanently restore cardiac function after acute or chronic ischemia. Improved understanding of the basic cellular and molecular mechanisms underlying the cardiac injury response may reveal novel therapeutic targets for treating heart disease.

In the 1st wk of life, neonatal mice can achieve full cardiac functional recovery after acute injury, such as coronary artery occlusion or apex resection; however, this transient regenerative capacity is lost after postnatal day seven (Porrello et al, 2011). The loss of regenerative potential is because of a decrease in cardiomyocyte proliferation and the subsequent formation of a fibrotic scar as an imperfect and poorly functional replacement (Song et al, 2012; Porrello et al, 2013; Senyo et al, 2013; Chong et al, 2014; Kong et al, 2014; van Berlo & Molkentin, 2014; Foglia & Poss, 2016; Travers et al, 2016; Deshmukh et al, 2019). Although most research efforts have focused on cardiomyocyte replenishment as the therapeutic remedy for combating heart disease, recent studies have uncovered a critical role for noncardiomyocytes in modulating the local environment to support regeneration (Aurora et al, 2014; Lavine et al, 2014).

The cardiac injury response is a highly dynamic and complex multicellular process in which noncardiomyocyte cell types such as immune cells and ECs play crucial roles to aid in recovery of the ischemic heart (Aurora et al, 2014; Lavine et al, 2014; Bajpai et al, 2018, 2019; Liao et al, 2018; Nahrendorf, 2018; Das et al, 2019; Dick et al, 2019; Lai et al, 2019). During acute myocardial infarction (MI), different cell types including cardiomyocytes, cardiac fibroblasts, and ECs promote changes in the cardiac immune environment to facilitate repair (Rainer et al, 2014; Frieler & Mortensen, 2015). The immune environment during the cardiac injury response is essential for debris clearance, inflammation resolution, and angiogenesis (Lai et al, 2019). Although studies on ECs have largely focused on their roles in angiogenesis and vascularization (Marín-Juez et al, 2016; Ingason et al, 2018; Das et al, 2019; Lu et al, 2021), ECs can also modulate their local immune environment (Seternes et al, 2002; Ding et al, 2012; Wohlleber & Knolle, 2016; Qiu et al, 2018; Amersfoort et al, 2022). Improved understanding of the molecular regulation of ECs and immune modulation during the cardiac injury response is essential for effective therapeutic development against heart disease.

Here, we provide evidence of an EC-specific immune regulatory signature in chronic human heart disease obtained by performing imaging mass cytometry (IMC) of pediatric heart failure (HF) tissues and computational analysis of adult HF single-cell transcriptomic datasets. To further dissect the role of EC-mediated immune regulation in the cardiac injury response, we performed single-cell profiling of regenerative and nonregenerative murine MI models. In regenerative-stage hearts, we observed EC-specific transcriptomic changes associated with cell proliferation, the IFN response, and immune regulation. Furthermore, we identified distinct subpopulations of ECs enriched for these gene signatures. Binding motifs of IFN-responsive transcription factors (TFs), IRF7, BATF2, and STAT1, were enriched near accessible chromatin regions of immune regulatory genes, highlighting potential transcriptional regulators of EC immune regulatory function during the cardiac injury response. These findings support that EC proliferation and immune cell crosstalk occur in response to IFN signaling during the cardiac injury response. The observation of these features in both human HF and murine MI models suggests that the immune regulatory function of ECs may be involved in resolving cardiac stress induced by both acute injury in MI and adverse remodeling in chronic HF.

## Results

### Human heart failure tissues exhibit EC-specific immune regulatory signatures

**Figure 1.**
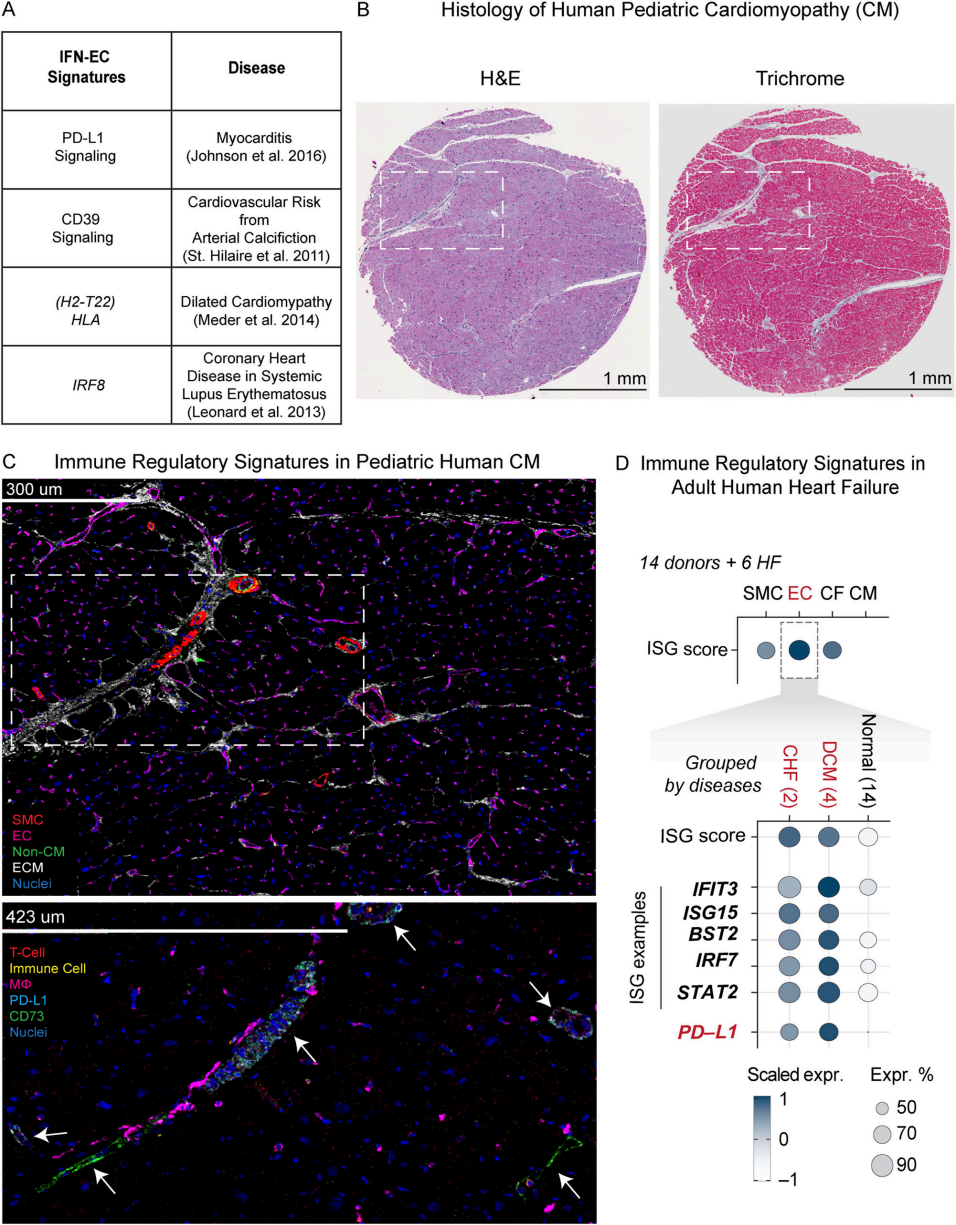
IFN-responsive and immune regulatory signaling by endothelial cells in human heart failure tissues. Human heart failure tissues exhibit EC-specific immune regulatory signatures. **(A)** Table of genes implicated in human heart disease. **(B)** Hematoxylin and eosin (left) and trichrome (right) staining of cardiac tissue from a pediatric patient diagnosed with dilated cardiomyopathy (DCM). **(B, C)** Imaging mass cytometry showing the expression of the selected markers in pediatric DCM cardiac tissue shown in panel (B). The lower panel is a zoomed-in view of the region inside the dashed box in the upper panel. Arrows indicate the expression of PD-L1 (blue) and CD73 (green) in vessels with nearby macrophage infiltration. **(D)** Dot plot showing the IFN-stimulated gene score and expression of IFN response and immune regulatory gene signatures in cardiac endothelial cells (ECs) from adult patients with DCM or congestive heart failure compared with those from normal donors.

**Figure S1.**
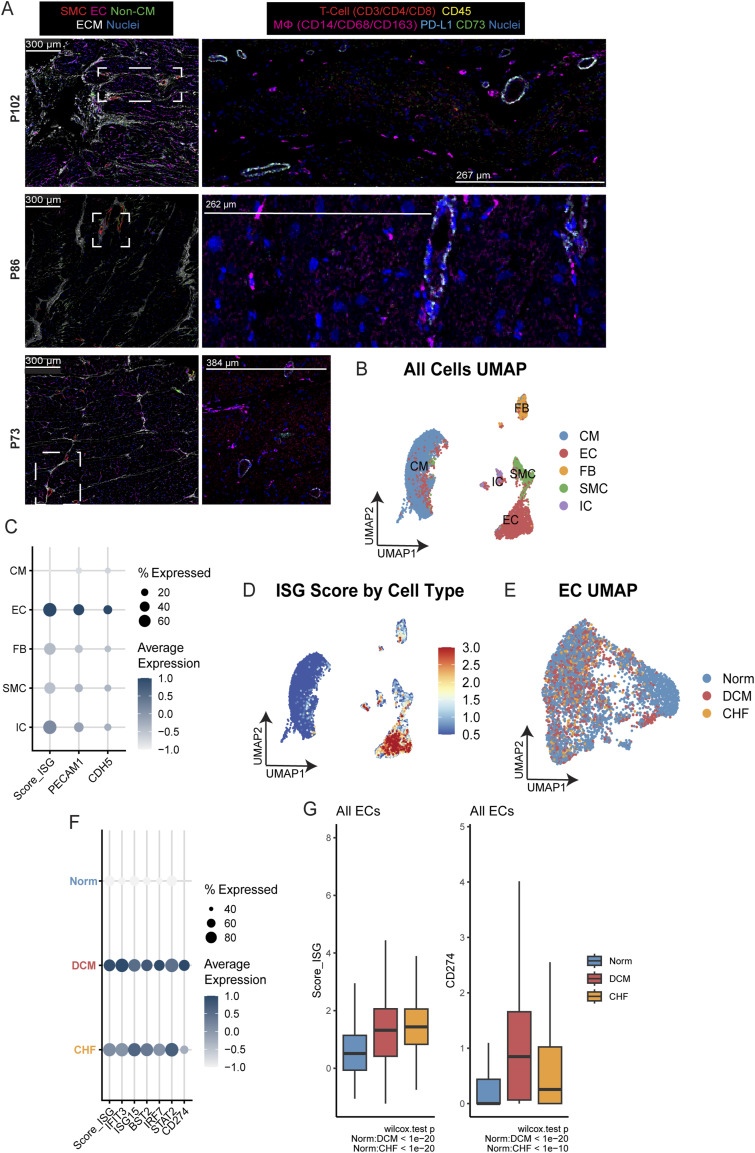
IFN-EC signatures in human heart disease. **(A)** Imaging mass cytometry showing expression of the indicated markers in cardiac tissue from pediatric patients with heart failure. In this context, CD45 staining (yellow) did not correlate with the more robust expression of macrophage markers. Dashed boxes indicate the zoomed-in region that is highlighted. **(B)** UMAP of cardiac cell clusters identified in the snRNA-sequencing data of human heart failure tissues. **(C)** Dot plot showing relative IFN-stimulated gene (ISG) score and expression of endothelial genes in different cardiac cell clusters. **(D)** UMAP showing ISG score by cell type. **(E)** UMAP showing the distribution of diseased versus donor cardiac cells. **(F)** Dot plot showing ISG score and ISG genes in diseased and donor cardiac cells. **(G)** Quantification of ISG score (left) and CD274 (right) expression in ECs from diseased and donor cardiac samples.

Previous studies have uncovered associations between immune-response genes (such as PD-L1, CD73, MHC-I, and IRF8) and human heart diseases including cardiomyopathy, atrial fibrillation, and atherosclerosis (Fig 1A) (Hennecke & Wiley, 2001; St Hilaire et al, 2011; Leonard et al, 2013; Meder et al, 2014; Johnson et al, 2016; Esfahani et al, 2019; Bracamonte-Baran et al, 2021). Moreover, recent findings of decreased macrophage proliferation in human adult and pediatric heart disease tissues suggest immune inhibitory signaling from cardiac cells in response to injury and stress (Hill et al, 2022; Koenig et al, 2022). To identify potential cell types that engage with the immune microenvironment in human heart disease, we performed IMC on pediatric HF samples. Tissue samples were collected from four pediatric patients with end-stage HF who were undergoing either ventricular assist device placement or heart transplantation (Hill et al, 2022). Patient diagnoses included hypertrophic obstructive cardiomyopathy and chemotherapy-induced cardiomyopathy (Fig 1B and Table S1). A panel of 23 markers, including proteins associated with immune cells, vessels, extracellular matrix, and immune inhibitory signaling molecules, was used to spatially resolve the cellular organization of these tissue samples (Table S2). IMC revealed the expression of PD-L1 and CD73, which functions in immunosuppressive and anti-inflammatory signaling, in vessels with perivascular macrophage infiltration (Figs 1C and S1A) (Chen et al, 2018; Eichin et al, 2021). This observation supports the hypothesis that ECs express anti-inflammatory signals that may dampen the local inflammatory microenvironment in pediatric HF.

Table S1. Patient information.

Table S2. Imaging mass cytometry antibodies.

To investigate whether EC-specific immune regulatory signatures exist in adult HF tissues, we reanalyzed published single-cell datasets from heart tissues of patients with dilated cardiomyopathy (DCM), congestive HF or no HF (Calcagno et al, 2020; Wang et al, 2020). Our analysis revealed that *CD274* (PD-L1) was enriched in adult heart failure tissues compared with non-HF tissues (Figs 1D and S1F and G). Because the expression of PD-L1 and CD73 on ECs is associated with type 1 and type 2 IFN signaling in other contexts (Lucas et al, 2018; Eichin et al, 2021), we developed an IFN-stimulated gene (ISG) score to identify cells transcriptionally responsive to IFN stimulus (Fig S1D). The ISG score is determined using several well-characterized and canonical targets of IFN signaling, including, *BST2*, *IRF7*, and *STAT2*. We found that ECs from HF tissues had higher ISG scores than ECs from non-HF tissues (Figs 1D and S1E–G; Table S3). These data show that ECs express IFN and immune regulatory signatures to interact with the cardiac immune environment in both pediatric and adult human HF. Together, these findings suggest that EC-mediated immune regulation may play a role during the stress response to chronic inflammation and adverse remodeling in human HF.

Table S3. Genes used for IFN-stimulated gene scoring.

### Single-cell transcriptomic analysis reveals an immune-responsive gene signature in endothelial cells from regenerative hearts after myocardial infarction

**Figure 2.**
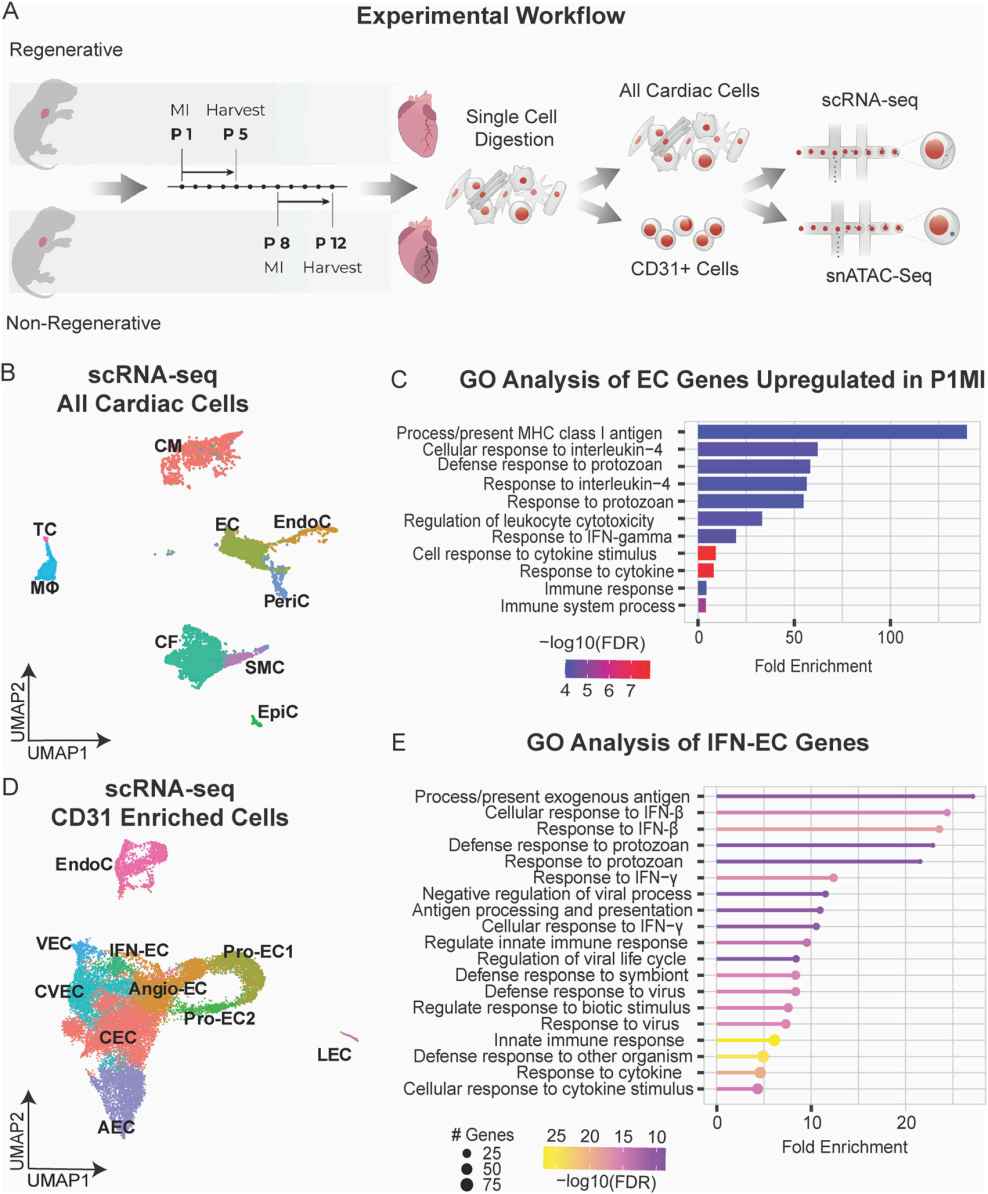
Single-cell RNA sequencing (scRNA-seq) of all cardiac cells and CD31+-enriched cells from regenerative- and nonregenerative-stage murine hearts after myocardial infarction (MI). scRNA-seq of ECs reveal a subpopulation enriched with immune responsive signatures. **(A)** Schematic showing the experimental design for the scRNA-seq and single-nuclei ATAC sequencing analysis of regenerative mouse hearts (MI at P1 and harvested at P5) and nonregenerative mouse hearts (MI at P8 and harvested at P12). **(B)** UMAP representation of cardiac cell clusters identified from scRNA-seq analysis. Clusters were color coded according to cell types (left). CM, cardiomyocytes; EC, endothelial cells; CF, fibroblasts; PeriC, pericytes; SMC, smooth muscle cells; EndoC, endocardial cells; EpiC, epicardial cells; MF, macrophages; TC, T-cells. **(C)** Gene ontology (GO) analysis of differentially expressed genes up-regulated in ECs of regenerative-stage hearts versus nonregenerative-stage hearts after MI. **(D)** UMAP representation of CD31+-enriched cell clusters from single-cell RNA sequencing (scRNA-seq), color coded according to cell type. CEC, capillary endothelial cell; Angio-EC, angiogenic endothelial cell; Pro-EC1, proliferative endothelial cell 1; Pro-EC2, proliferative endothelial cell 2; IFN-EC, interferon capillary endothelial cell; CVEC, capillary venous endothelial cell; VEC, venous endothelial cell; AEC, arterial endothelial cell; LEC, lymphatic endothelial cell; EndoC, endocardial cell. **(E)** Gene ontology (GO) analysis of differentially expressed genes up-regulated in the IFN-EC cluster of regenerative-stage hearts versus nonregenerative-stage hearts after MI.

**Figure S2.**
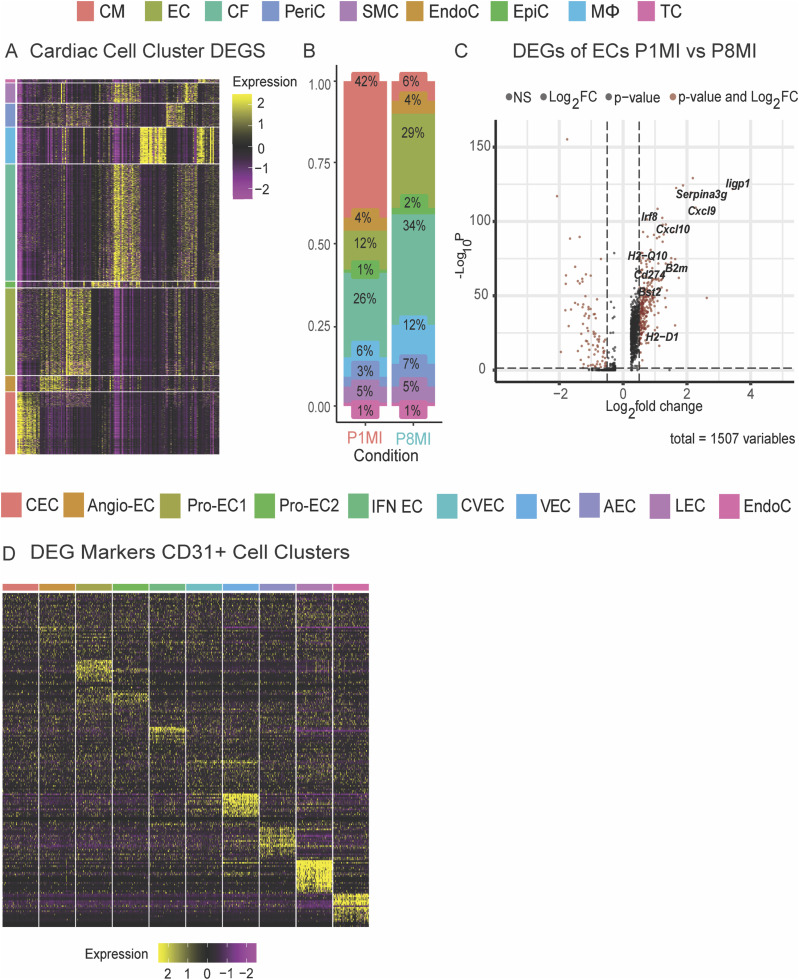
Single-cell profiling of murine hearts 4 d after myocardial infarction (MI) at P1 and P8. **(A)** Heatmap representation of cardiac cell clusters from single-cell RNA sequencing (scRNA-seq) data. Clusters are color coded for cardiomyocyte (CM), endothelial (EC), fibroblast (CF), pericytes (PeriC), smooth muscle cell (SMC), endocardial (EndoC), epicardial (EpiC), macrophage (MΦ), and T-cell (TC) clusters. **(B)** Percentages of cells from each cluster for cardiomyocyte (CM), endothelial (EC), cardiac fibroblast (CF), pericyte (PeriC), smooth muscle cell (SMC), endocardial (EndoC), epicardial (EpiC), macrophage (MΦ), and T-cell (TC) clusters identified in scRNA-seq analysis. **(C)** Volcano plot showing differentially expressed genes in ECs from regenerative and nonregenerative hearts. **(D)** Heatmap representation of cardiac cell clusters from scRNA-seq data. Clusters are color coded for different cell types.

To investigate the role of EC-mediated immune regulation in facilitating cardiac repair, we used a murine model of regenerative and nonregenerative ischemic injury induced by left anterior descending artery occlusion (LAD-O) (Porrello et al, 2011). LAD-O was performed at postnatal day one (P1MI, regenerative stage) or eight (P8MI, nonregenerative stage), and cells were isolated from the left ventricle 4 d later at P5 or P12, respectively (Fig 2A). We then performed single-cell RNA sequencing (scRNA-seq) on the isolated cells to compare the transcriptomes between regenerative- and nonregenerative-stage neonatal mouse hearts at single-cell resolution. After discarding low-quality cells and doublets (see the Materials and Methods section and Table S4), we identified 11,333 cells that were separated into nine distinct clusters based on gene expression (Figs 2B and S2A and B). We manually annotated these clusters using well-defined gene expression signatures (Table S5) and identified cells corresponding to ECs, cardiomyocytes, cardiac fibroblasts, macrophages, T cells, pericytes, epicardial cells, endocardial cells, and smooth muscle cells (SMCs).

Table S4. Quality control of sequencing experiments. Quality control for single-cell RNA-sequencing experiments. Quality control for single-nuclei ATAC-sequencing experiments.

Table S5. Cell cluster markers from whole heart single-cell RNA sequencing.

Next, we explored EC-specific differences between the transcriptomes of regenerative- (P1MI) and nonregenerative-stage (P8MI) mouse hearts (Fig S2C and Table S6). We identified 1,728 differentially expressed genes (DEGs) (adjusted *P*-value < 0.01, log_2_ fold change ≥ 0.2) that were more highly expressed in regenerative-stage ECs than in nonregenerative-stage ECs (Table S6). To investigate potential pathways activated during cardiac repair, we performed gene ontology (GO) enrichment analysis using the 50 most significant DEGs (Ge et al, 2020). GO analysis revealed gene sets associated with cell proliferation (e.g., *Top2a*, *Ccnd1*), antigen presentation (e.g., *β2m*, *H2-Q4*), the IFN response (e.g., *Iigp1*, *Ifitm3*), and immune processes (e.g., *Serpina3g*, *Lgals9*) (Figs 2C and S2C and Table S6). These data reveal a novel EC-specific immune responsive gene signature that was not identified previously in the single-cell profiling data of regenerative- and nonregenerative-stage hearts (Wang et al, 2020). Taken together, our findings reveal that transcriptomic differences between regenerative- and nonregenerative-stage ECs are associated with cell proliferation, antigen presentation, the IFN response, and immune processes.

Table S6. Differentially expressed genes (DEGs) and differentially accessible regions (DARs) from whole heart single-cell RNA sequencing. DEGs up-regulated in P1MI. DEGs down-regulated in P1MI. DARs up-regulated in P1MI. DARs Down-regulated in P1MI. Overlapping DEGs and DARs up-regulated in P1MI.

### Cardiac EC-specific transcriptomic profiling reveals distinct EC states with cell cycle and IFN response signatures

**Figure S3.**
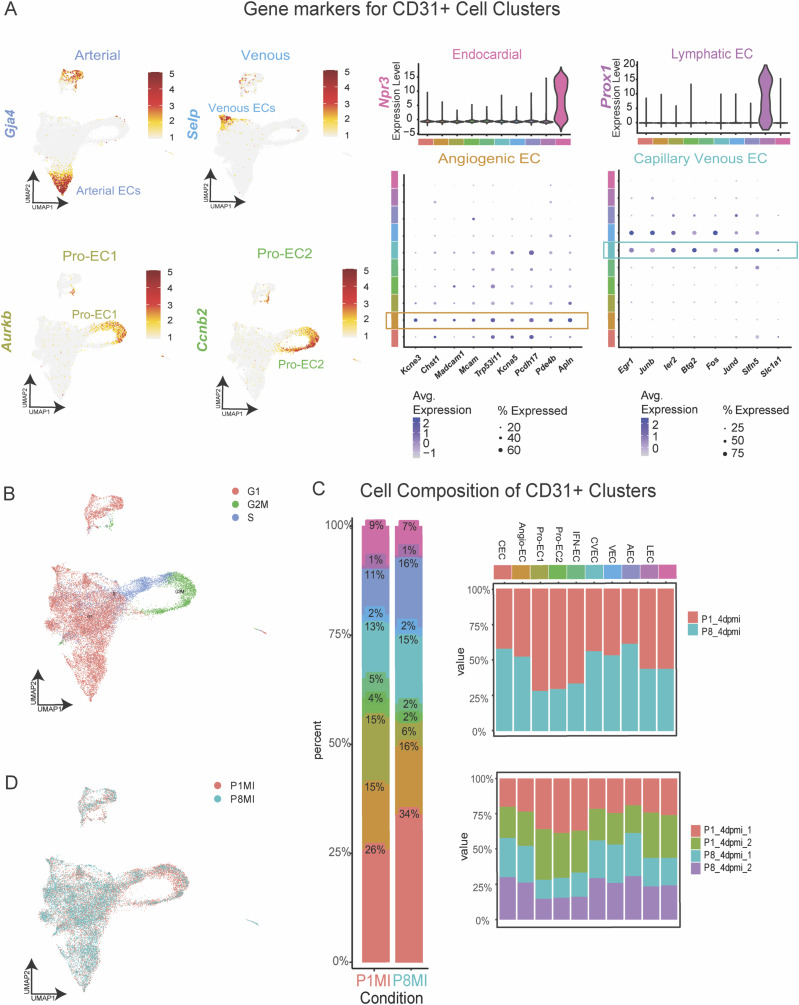
Characterization of CD31+-enriched cell clusters from murine hearts 4 d after myocardial infarction (MI) at P1 and P8. **(A)** Expression of marker genes for the indicated CD31+-enriched cell clusters. **(B)** UMAP representation of cell cycle scoring. **(C)** Composition plots showing the distribution of cell clusters in each condition (left) and the contribution of each condition to each cell cluster (right). CEC, capillary EC; Angio-EC, angiogenic EC; Pro-EC1, proliferative EC 1; Pro-EC2, proliferative EC2; IFN-EC, interferon capillary EC; CVEC, capillary venous EC; VEC, venous EC; AEC, arterial EC; LEC, lymphatic EC; EndoC, endocardial cells. **(D)** UMAP representation of experimental conditions.

To further study the transcriptomic heterogeneity of ECs from regenerative-stage and nonregenerative-stage hearts in greater detail, we performed scRNA-seq of ECs (CD31^+^) enriched from murine hearts injured during regenerative (P1MI) and nonregenerative stages (P8MI) using magnetic-activated sorting (MACs) (Fig 2A and see the Materials and Methods section). After filtering out low-quality cells and doublets (see the Materials and Methods section), we detected 21,414 cells that were clustered into 10 unique CD31^+^ subpopulations (Fig 2D). The 10 distinct clusters were transcriptionally heterogeneous (Fig S2D, Table S7). Using established marker gene expression (Schiller et al, 2019; Goveia et al, 2020; Kalucka et al, 2020; Travaglini et al, 2020; Schupp et al, 2021), we identified arterial EC, capillary EC, capillary venous EC, venous EC, angiogenic EC, lymphatic EC, and endocardial cell clusters (Fig S3A and Table S7). In addition, we identified three specialized EC clusters (Figs 2D and S2D, Table S7).

Table S7. CD31-enriched cells. Cell cluster markers from CD31-enriched single-cell RNA sequencing (scRNA-seq). Statistics for cellular composition comparison between P1MI and P8MI. Genes enriched in the Pro-EC1 Cluster. GO analysis of genes enriched in the Pro-EC1 Cluster. Genes enriched in the Pro-EC2 Cluster. GO analysis of genes enriched in Pro-EC2 Cluster. Genes enriched in IFN-EC Cluster. GO analysis of genes enriched in IFN-EC Cluster. Overlapping genes enriched in IFN-EC Cluster and up-regulated in P1MI from whole heart scRNA-seq. Transcription factors enriched in CD31-enriched cell clusters. Cell cluster markers from CD31-enriched scRNA-seq including immune cells.

**Figure 3.**
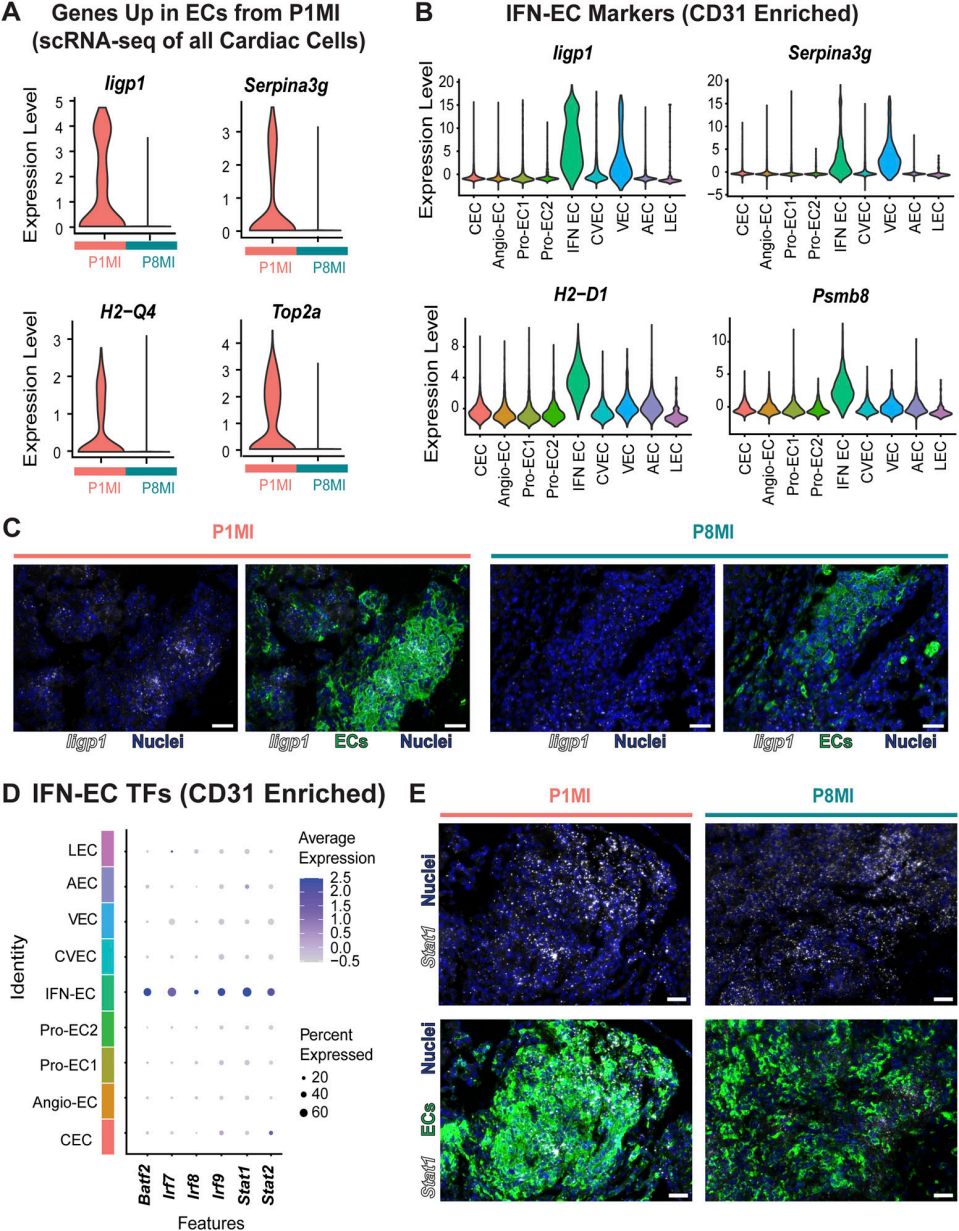
Single-cell transcriptomic analysis reveals an immune responsive gene signature in endothelial cells from regenerative-stage mouse hearts after myocardial infarction (MI). Single-cell RNA sequencing of ECs reveal a subpopulation enriched with immune responsive signatures. **(A)** Violin plots showing expression levels of the indicated genes in endothelial cells (ECs) from single-cell RNA sequencing data of all cardiac cells. **(B)** Violin plots showing expression levels of indicated genes in the CD31^+^ cell cluster. **(C)** Representative RNA-scope images showing RNA expression of the indicated genes in ECs of regenerative-stage P1MI (left) and nonregenerative-stage P8MI (right) hearts. **(D)** Dot plot showing gene expression levels of the indicated transcription factors in each CD31^+^ cell cluster. **(E)** Representative RNA-scope images showing RNA expression of the indicated transcription factors in ECs of regenerative-stage P1MI (left) and nonregenerative-stage P8MI (right) hearts.

To further explore the characteristics of the three specialized EC clusters, we identified the DEGs that distinguish the three clusters (adjusted *P* < 0.01, log_2_ fold change ≥ 1.5). We also examined the enriched GO terms for each cluster (Fig 2E, Table S7). All three clusters were enriched with distinct cell cycle IFN-response signatures (e.g., *Iigp1*, *Ifitm3*) that corresponded with the genes more highly expressed in regenerative-stage than in nonregenerative-stage ECs from our previous whole heart dataset (Figs 2C and 3A, Table S6). Two of the clusters were enriched in cell cycle processes and were therefore annotated as proliferating EC clusters ProEC-1 and ProEC-2 (Fig S3A and Table S7). Cell cycle analysis of ProEC-1 and ProEC-2 cells revealed an enrichment of cells in S or G2M phases, respectively (Fig S3B). In addition, ProEC-1 and ProEC-2 were most closely associated with angiogenic ECs, which is consistent with EC proliferation observed during angiogenesis and vessel growth in regenerating hearts (Schaper, 1996; Dor & Keshet, 1997; Ware & Simons, 1997; Das et al, 2019). The remaining specialized EC cluster, which appeared similar to capillary ECs, showed specific enrichment of IFN-γ signaling (e.g., *Iigp1*, *Ifitm3*) and antigen-presentation pathways (e.g., *H2-D1*, *H2-K1*, *Psmb8*) and was termed the IFN-EC cluster (Figs 2E and 3B, and Table S7). RNA-scope imaging showing RNA expression of *Iigp1* in ECs of regenerative-stage heart tissue supports observations from both scRNA-seq datasets (Fig 3C). Furthermore, the IFN-EC cluster featured higher expression of IFN-associated TFs such as *Batf2*, *Irf7*, *Irf8*, *Irf9*, *Stat1*, and *Stat2* than did most other EC populations (Fig 3D). RNA-scope imaging of EC-specific *Stat1* RNA expression in regenerative-stage hearts supports this observation (Fig 3E).

We next used the CD31+-enriched datasets and compared cell composition between regenerative-stage (P1MI) and nonregenerative-stage (P8MI) mouse hearts after ischemic injury. The Pro-EC and IFN-EC populations were significantly enriched in regenerative-stage hearts (*P* < 0.001, log fold difference > 1; Fig S3C and D, Table S7). Therefore, consistent with our scRNA-seq dataset revealing an up-regulation of cell cycle, IFN response, and immune regulatory genes in the ECs of regenerative-stage hearts versus nonregenerative-stage hearts after ischemic injury (Figs 2C and 3A), we highlight distinct EC subpopulations with these gene signatures (Fig 3B). Our data also suggest that the unique IFN-EC cluster, in addition to the two proliferating EC clusters, may be important for the regenerative response after MI.

### IFN-responsive TF binding motif networks are enriched at IFN-EC genes

**Figure 4.**
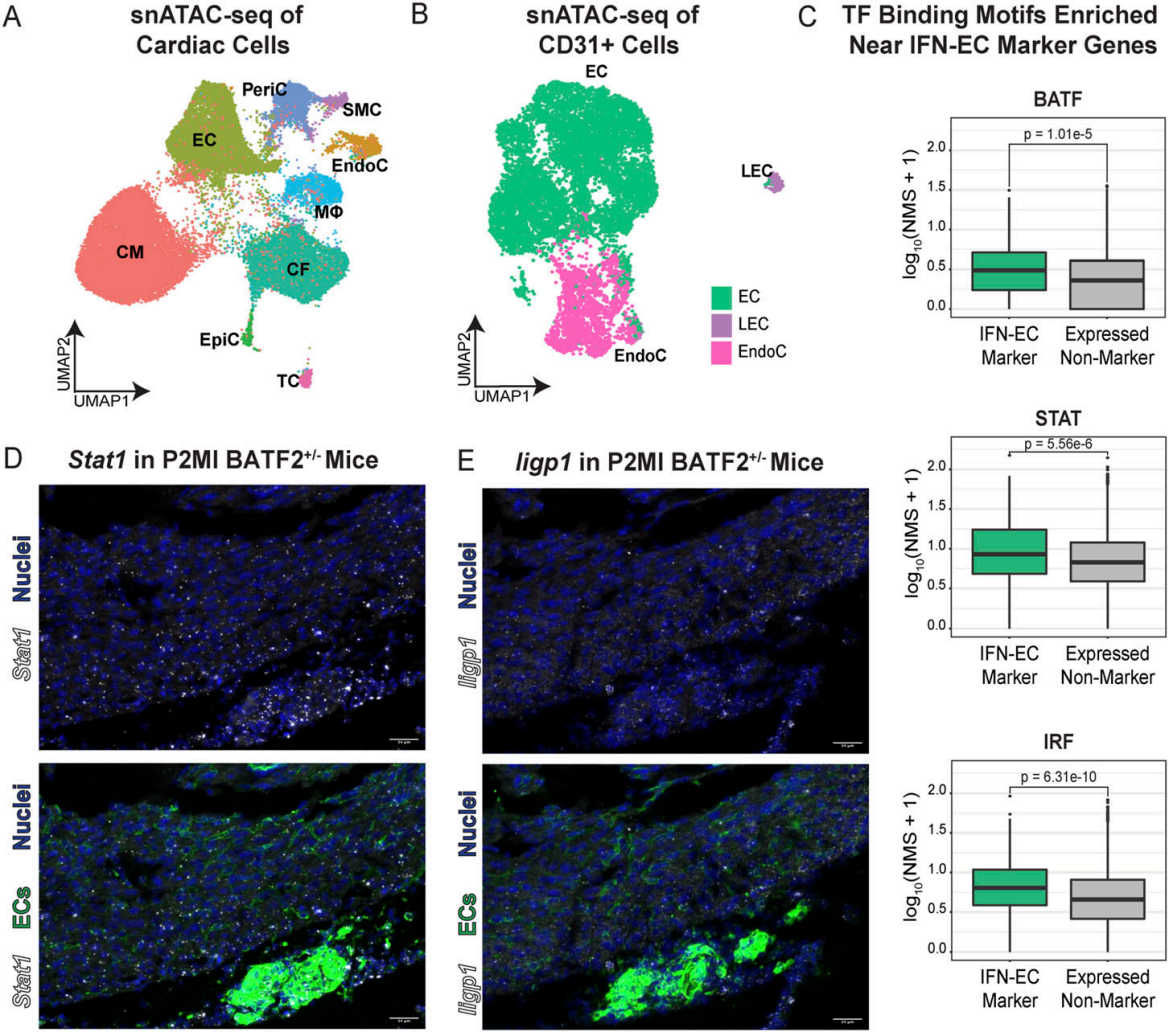
Single-nuclei chromatin accessibility profiling of cardiac cells from regenerative- and nonregenerative-stage mouse hearts after myocardial infarction (MI). Single-nuclei ATAC sequencing (snATAC-seq) of ECs reveal potential transcription factor regulators of IFN-EC gene signature. **(A)** UMAP representation of cardiac cell clusters from snATAC-seq data. Clusters are color coded according to cell type. EC, endothelial cells; LEC, lymphatic endothelial cell; EndoC, endocardial cells. **(B)** UMAP representation of CD31^+^ cell clusters from snATAC-seq data. Clusters are color coded according to the cell type. **(C)** Normalized motif score plots showing the occurrence of binding motifs for the indicated transcription factors correlated with chromatin regions of IFN-EC differentially expressed (DE) differentially expressed genes versus non-differentially expressed genes. **(D)** Representative RNA-scope images showing *Stat1* RNA expression in ECs of *BATF*^*+/−*^ knockout mouse hearts. **(E)** Representative RNA-scope images showing *Iigp1* RNA expression in ECs of *BATF*^*+/−*^ knockout mouse hearts.

**Figure S4.**
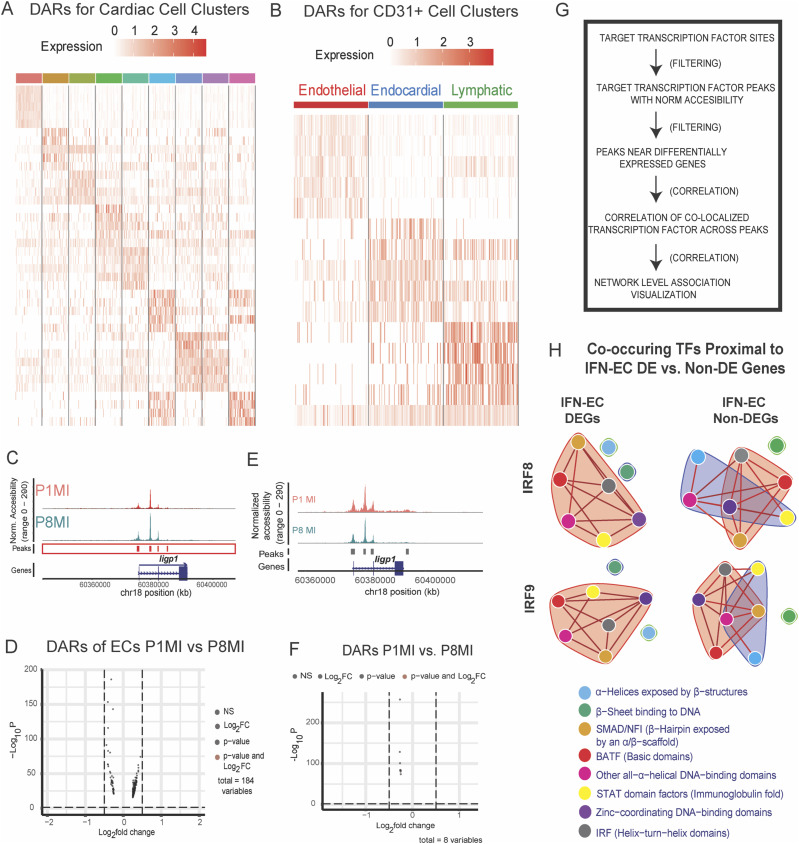
Single-nuclei assay for transposase-accessible chromatin followed by single-nuclei ATAC sequencing (snATAC-seq) of CD31+-enriched cells from murine hearts 4 d after myocardial infarction at P1 and P8. **(A)** Heatmap representation of cardiac cells identified from snATAC-seq data. Cells are color coded according to clusters. **(B)** snATAC-seq tracks of chromatin accessibility for the indicated gene in regenerative-stage and nonregenerative-stage cardiac endothelial cells (ECs). **(C)** Volcano plot showing differentially accessible regions in endothelial cells (ECs) from regenerative and nonregenerative hearts. **(D)** Heatmap representation of CD31^+^ cell clusters identified from snATAC-seq data. Clusters are color-coded according to different cell types. **(E)** Coverage plot showing chromatin accessibility of the indicated IFN-EC differentially expressed genes (DEGs) in CD31^+^ clusters. **(F)** Volcano plot showing differentially accessible regions in ECs from regenerative and nonregenerative hearts. **(G)** Workflow describing the pipeline for network analysis of co-occurring transcription factors (TFs) in accessible chromatin regions of genes of IFN-EC DEGs versus non-DEGs. **(H)** Network plots showing TF families co-occurring with the indicated TFs nearby chromatin regions of IFN-EC DEGs versus non-DEGs. Edges indicate neighborhoods of likely co-occurring TFs. Each TF family is color-coded according to the legend.

To map the chromatin accessibility landscape in regenerative- and nonregenerative-stage mouse hearts under ischemic stress, we performed single-nuclei assay for transposase-accessible chromatin followed by single-nuclei ATAC sequencing (snATAC-seq) on nuclei isolated from regenerative- and nonregenerative-stage mouse hearts after LAD-O (P1MI and P8MI) (Fig 2A). After removing low-quality nuclei (see the Materials and Methods section and Table S3), 37,881 nuclei remained, with 120,401 accessible chromatin regions identified. Using scRNA-seq data to inform cluster identification (Fig 2B) in the snATAC-seq data, we identified nine clusters representing ECs, CMs, cardiac fibroblasts, macrophages, T cells, pericytes, epicardial cells, endocardial cells, and SMCs (Figs 4A and S4A).

To explore potential EC-specific changes in chromatin accessibility, we also performed snATAC-seq on CD31-enriched cells from regenerative- and nonregenerative-stage mouse hearts harvested 4 d after injury at P1 and P8 (Fig 2A). A total of 11,289 cells with 100,155 open chromatin regions were obtained. Label transferring from the scRNA-seq data (Fig 2C) identified and annotated three distinct clusters as EC, EndoC, and lymphatic endothelial cell (LEC) (Figs 4B and S4B).

We investigated whether chromatin accessibility differences underlie the difference in gene expression between regenerative- and nonregenerative-stage ECs. Although we did not observe significant chromatin accessibility differences (adjusted *P* < 0.01, log_2_ fold change ≥ 0.5) associated with IFN-EC DEGs, such as *Iigp1* and *H2-D1* (Fig S4C–F and Table S6), changes in chromatin accessibility do not always accompany changes in gene expression. This has been seen in some contexts of IFN signaling in which other mechanisms of regulation, such as histone modifications, may play a role (Hogan et al, 2017; Kamada et al, 2018; Hota et al, 2022; Platanitis et al, 2022). Because gene expression is regulated by the combinatorial binding of TFs to accessible chromatin (Felsenfeld et al, 1996; Thurman et al, 2012; Tsompana & Buck, 2014; Klemm et al, 2019), we focused on the TF motifs in accessible chromatin that may be regulating transcription. We first developed a normalized motif score (NMS) for every gene using the three classes of TFs that were enriched in the IFN-EC cluster (Fig 3D), namely the IRFs, BATFs, and STATs (see the Materials and Methods section). When applying the NMS for the IRFs, BATFs, and STATs to the IFN-EC marker genes, we found that the NMS was significantly higher for all three TF families at IFN-EC marker genes than at other expressed genes (Fig 4C and Table S8). RNA-scope imaging of cardiac tissue from mice with the heterozygous knockout of *BATF2* (*BATF*^*+/−*^) suggest a decrease in *Iigp1* and *Stat1* (IFN-EC genes) RNA expression (Fig 4D and E). Together, these data suggest that IRFs, BATFs, and STATs may be involved with the transcription of IFN-EC genes.

Table S8. Transcription factor-binding motifs enriched in regenerative-stage ECs from whole-heart single-cell RNA sequencing. All activators. Strong activators.

We then used network analysis to identify DNA-binding motifs of all TF superclasses that co-occur with IRF motifs within accessible chromatin regions of IFN-EC marker genes (Fig S4G). Spectral network analysis classified the immunoglobulin fold superclass, basic domain class, and helix-turn-helix domain class TFs, which includes the STATs, BATFs, and IRFs, respectively, as members of the same community for IFN-EC marker genes, that is, these factors co-occur in more similar patterns (Fig S4E). However, STATs and BATFs no longer formed communities with IRFs within the accessible chromatin regions at other expressed genes, supporting that the co-occurrence of TFs enriched in IFN-ECs (i.e., BATF2, IRF7/8/9, and STAT1/2) at accessible chromatin regions of IFN-EC marker genes may be important for their transcription.

### IFN-ECs express genes encoding immune regulatory ligands during cardiac regeneration

**Figure S5.**
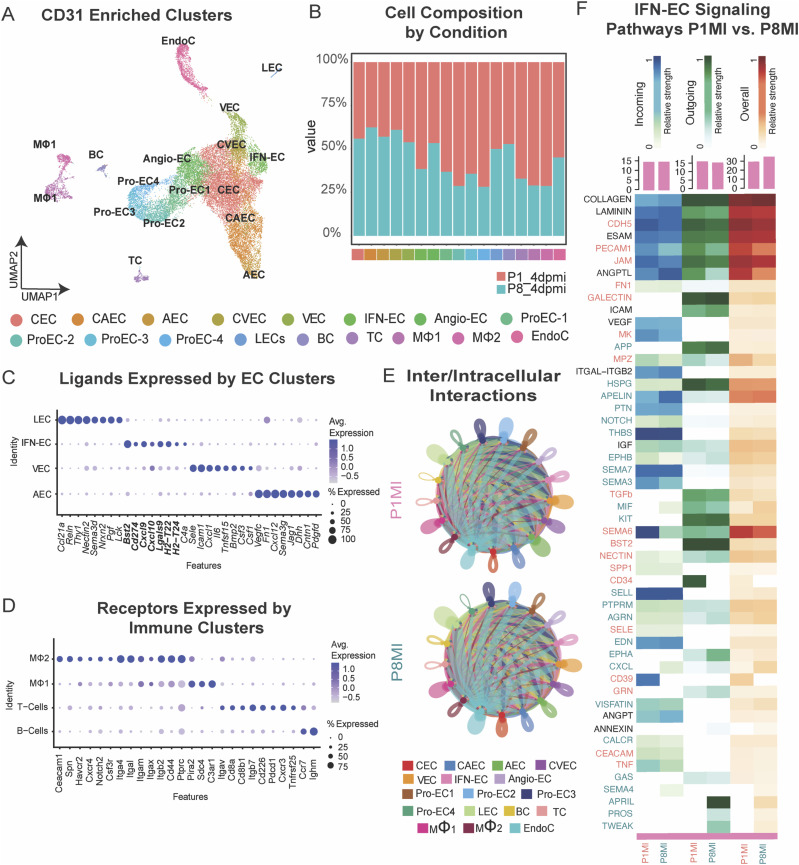
Ligand–receptor analysis of CD31+-enriched cells from regenerative and nonregenerative hearts. **(A)** UMAP representation of CD31+-enriched cell clusters identified from single-cell RNA sequencing data. Cells are color coded according to different cell types. **(B)** Composition plots showing the contribution of each condition to each cell cluster. **(C)** Dot plot showing differentially expressed ligands in the selected EC clusters. **(D)** Dot plot showing immune cell expression of receptors for endothelial cell (EC) ligands. **(E)** Chord plots showing the number of intercellular and intracellular interactions in regenerative and nonregenerative hearts. **(F)** Heatmap showing the incoming, outgoing, and overall signaling pathways for IFN-ECs from regenerative and nonregenerative hearts. CEC, capillary EC; CAEC, capillary arterial EC; AEC, arterial EC; CVEC, capillary venous EC; VEC, venous EC; Angio-EC, angiogenic EC; IFN-EC, interferon capillary EC; Pro-EC1, proliferative EC 1; Pro-EC2, proliferative EC2; Pro-EC3, proliferative EC3; Pro-EC4, proliferative EC4; LEC, lymphatic EC; BC, B-cells; TC, T-cells; MΦ1, macrophage 1; MΦ2, macrophage 2; EndoC, endocardial cells.

**Figure 5.**
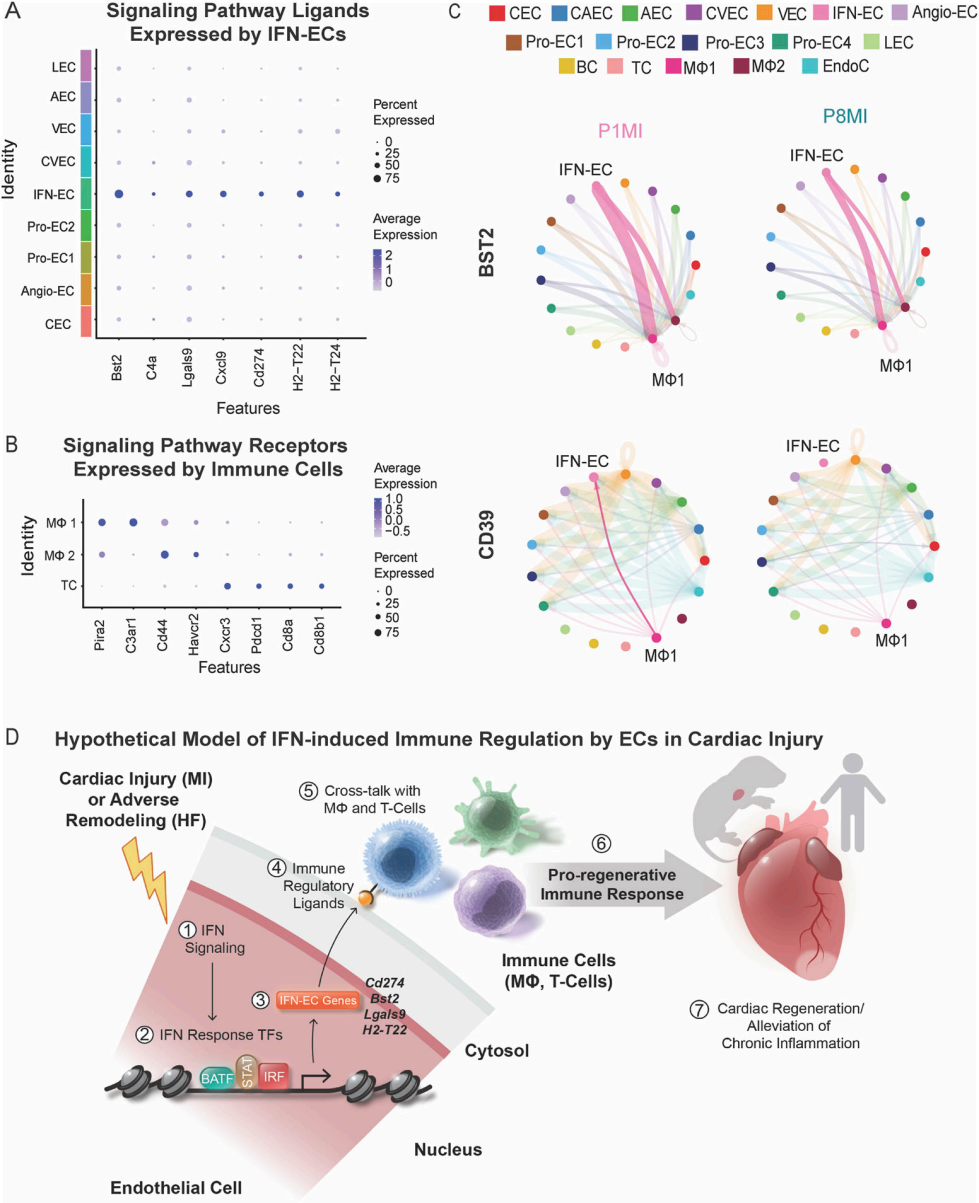
Ligand-receptor analysis of CD31-enriched cells from regenerative-stage and nonregenerative-stage mouse hearts after myocardial infarction (MI). EC subpopulations express genes encoding immune regulatory ligands. **(A)** Dot plot showing the indicated ligands enriched in the IFN–endothelial cell (EC) cluster. **(B)** Dot plot showing immune cell expression of receptors for ligands expressed in the IFN-EC cluster. **(C)** Chord plots showing the indicated signaling pathway network between the IFN-EC cluster and macrophages from regenerative-stage and nonregenerative-stage mouse hearts after MI. CEC, capillary endothelial cell; CAEC, capillary arterial endothelial cell; AEC, arterial endothelial cell; CVEC, capillary venous endothelial cell; VEC, venous endothelial cell; IFN-EC, interferon capillary endothelial cell; angio-EC, angiogenic endothelial cell; Pro-EC1, proliferative endothelial cell 1; Pro-EC2, proliferative endothelial cell 2; Pro-EC3, proliferative endothelial cell 13; Pro-EC4, proliferative endothelial cell 4; LEC, lymphatic endothelial cell; BC, B-cells; TC, T-cells; MF1, macrophage 1; MF2, macrophage 2; EndoC, endocardial cells. **(D)** Hypothetical model of IFN-induced immune regulation by ECs in cardiac injury.

Because IFN-ECs appear to be involved in immune signaling like the ECs in human HF tissues (Fig 1), we investigated the ligands expressed by IFN-ECs. By used the immune cells captured during CD31 enrichment, we characterized the signaling networks between ECs and immune cells of regenerative- and nonregenerative-stage mouse hearts after ischemic injury (Fig S5A and B). Examination of the IFN-EC marker genes revealed several signaling ligands with corresponding receptors on immune cells (Fig S5C and D). In IFN-ECs, enriched ligands included *Bst2*, *Cd274*, *Cxcl9*, *Lgals9*, *H2-T22*, *H2-T24*, and *C4a* (Fig 5A). *Bst2*, *Cd274*, and *Lgals9*, which are ligands for BST2, PD-L1, and GALECTIN signaling pathways, respectively, are related to type I IFN signaling and have immunosuppressive functions (Cao et al, 2009; Wu et al, 2014; Chen et al, 2018). Some receptors for the IFN-EC–enriched ligands, specifically *Pira2*, *C3ar1*, *Cd44*, and *Havcr2* (receptors for *Bst2*, *C4a*, and *Lgals9*, respectively), are expressed on macrophages (Fig 5B). In addition, some receptors for the IFN-EC–enriched ligands, specifically *Cxcr3*, *Pdcd1*, and *Cd8a*/*Cd8b1* (receptors for Cxcl9, Cd274, *H2-T22*, and *T2-T24*, respectively), are expressed on T cells (Fig 5B).

As a secondary approach, we used CellChat to identify ligand–receptor interactions between IFN-ECs and other cardiac cell types that were enriched in regenerative-stage mouse hearts compared with nonregenerative-stage hearts (Fig S5E and F) (Jin et al, 2021). Our analysis revealed that BST2 signaling, which is related to type I IFN signaling and inhibits the production of IFNs and pro-inflammatory cytokines from immune cells, is increased between IFN-ECs and macrophages in regenerative-stage hearts (P1MI) compared with nonregenerative-stage hearts (P8MI) (Fig 5C) (Cao et al, 2009). We also found that the CD39 signaling pathway, which is implicated in immunosuppression in cancer and myocardial protection after ischemic injury, is preferentially enriched between IFN-ECs and immune cells in regenerative-stage mouse hearts (P1MI) and signals from macrophages to IFN-ECs (Figs 5C and S5F) (Köhler et al, 2007; Saldanha-Araujo et al, 2011; Wheeler et al, 2012; Allard et al, 2017). These findings highlight transcriptomic changes associated with immunosuppressive signaling from ECs in regenerative-stage hearts.

## Discussion

The balance between activation and attenuation of inflammation during the injury response is critical for cardiac regeneration (Steffens et al, 2022). In human HF tissues, we uncovered an EC-specific IFN response and immune regulatory signature. IFN response and immune regulatory signatures in ECs have been described across different organs, but not in the context of cardiac regeneration after ischemic injury (Seternes et al, 2002; Qiu et al, 2018; Goveia et al, 2020; Kalucka et al, 2020; Amersfoort et al, 2022). Using single-cell transcriptomic profiling of cardiac ECs from regenerative-stage mouse hearts subjected to cardiac injury, we identified a subpopulation of capillary ECs, namely IFN-ECs, that showed a gene expression signature similar to that of ECs in human HF tissues (Fig 5D). These data revealed an enrichment of transcripts encoding IFN response TFs and immune regulatory ligands in IFN-ECs. Furthermore, an enrichment of binding motifs for IFN response TFs were enriched in IFN-EC markers genes, suggesting that these TFs may be upstream transcriptional activators of the immune regulatory signatures in ECs (Fig 5D). Together, our data hypothesizes a model in which EC-specific immune regulation be part of a central biologic response to acute and chronic cardiac inflammation induced by MI and HF, respectively (Fig 5D).

The response of the immune system to ischemic cardiac injury has been described as both beneficial in the initial phase and deleterious in the long-term injury response (Rurik et al, 2021). Inflammatory immune cells are important for debris clearance in the early stages of cardiac injury but can contribute to chronic inflammation and progressive pathologic remodeling in human HF. Therefore, regulation of the cardiac immune environment to maintain an immunosuppressed state may be an adaptive response in human HF, although ultimately an inadequate one.

Our IMC data of pediatric HF tissue revealed expression of CD274 (PD-L1) and CD73 (part of the CD39/CD73 signaling pathway) in ECs with immune infiltration. scRNA-seq analysis of adult HF tissues revealed a similar enrichment in ECs of *CD274* compared with those of donor tissues. Furthermore, this signature was accompanied by the expression of IFN-responsive TFs *IRF7*, *STAT2*, and *BST2*. Previous studies have shown that the inhibition of PD-L1 (CD274) signaling to treat cancer leads to subsequent myocarditis in humans, which can be alleviated with immunosuppression (Johnson et al, 2016; Mahmood et al, 2018; Moslehi et al, 2018; Salem et al, 2018; Esfahani et al, 2019). Furthermore, the expression of PD-L1 is important for engraftment after transplantation (Bracamonte-Baran et al, 2021). Mutations in *NT5E*, which encodes CD73, have been identified in patients with arterial calcification, an indicator of cardiovascular risk and inflammation (Lehto et al, 1996; Shaw et al, 2003; St Hilaire et al, 2011). These data, in addition to our findings, suggest that immunosuppressive signaling via PD-L1 and CD39/CD73 pathways by ECs may limit the deleterious effects of adverse inflammation in HF.

The datasets we obtained from murine models of MI revealed a gene expression signature characterized by the up-regulation of cell cycle, IFN response, and immune regulatory signatures in regenerative-stage hearts, suggesting that EC-mediated immune regulation, in addition to EC proliferation, is related to cardiac regeneration. Importantly, the up-regulation of immune responsive genes in regenerative-stage hearts may be attributed to a specific EC subpopulation, IFN-ECs. Our multi-omic analysis revealed the enrichment of transcripts and binding motifs of IFN response TFs, such as certain IRFs, BATFs, and STATs, in IFN-ECs and in regenerative-stage hearts, suggesting that these IFN response TF families may regulate the gene expression of IFN-EC marker genes. *Batf2* expression is related to type 1 and type 2 IFN signaling, modulates the immune response, and has been linked to *Irf* activity in immune cells (Schraml et al, 2009; Betz et al, 2010; Ise et al, 2011; Li et al, 2012; Jabeen et al, 2013; Roy et al, 2015; Matatall et al, 2016). *Irf7*, *8*, and *9* are important for immune regulation and are protective against various cardiac pathologies (Biron, 2001; Lohoff & Mak, 2005; Tamura et al, 2008; Döring et al, 2012; Jiang et al, 2014a, 2014b, 2014c; Zhang et al, 2014; McNab et al, 2015). STAT1 and STAT2 have been shown to bind IRF9 to form the trimer complex ISGF3, which binds the IFN-stimulated response element and induces transcription of genes such as *Irf7* (Honda & Taniguchi, 2006; Au-Yeung et al, 2013; Cheon et al, 2013; McComb et al, 2014). Importantly, we found *IRF7* to be enriched in ECs from adult HF tissues. Notably, gene variants in *IRF8* have been identified as risk factors for coronary heart disease (Leonard et al, 2013). These findings suggest that IFN-response TFs, which are typically active in immune cells, may regulate the transcription of a gene expression program promoting the IFN response and immune regulation in ECs.

Our analysis revealed that multiple cardiac EC populations express ligands involved in immune regulatory pathways. Studies have shown that LECs and venous ECs can participate in immune regulatory signaling with immune cells (Lee et al, 2014; Takeda et al, 2019; Brulois et al, 2020; Xiang et al, 2020). Furthermore, LECs can signal to macrophages, T cells, and dendritic cells via *Ccl21*, a lymphoid homing chemokine (Nagira et al, 1997; Willimann et al, 1998; Saeki et al, 1999; Luther et al, 2000; Shields et al, 2010; Card et al, 2014). Venous ECs have gene expression signatures associated with leukocyte recruitment and chemokine signaling (Thiriot et al, 2017; Goveia et al, 2020; Kalucka et al, 2020; Schupp et al, 2021). We found that the capillary EC subpopulation of IFN-ECs also expressed immune modulatory ligand genes such as *Bst2*, *Cd274*, *CD39*, and *H2-T22*. H2-T22 and H2-T24 are part of the MHC Class I family and are important for signaling to T cells (Hennecke & Wiley, 2001). Genes encoding MHC Class I and II proteins have been associated with a risk locus for DCM (Meder et al, 2014). Increased Bst2, Cd274, and CD39/CD73 signaling in regenerative stage hearts is consistent with our hypothesis that the EC-specific IFN response and immune regulation may mediate the immune in human HF and cardiac regeneration.

In conclusion, our findings reveal a novel role for ECs in immune regulation in human HF and in mice after cardiac ischemic injury (Fig 5D). Our single-cell sequencing datasets can be used for further study into EC function, signaling pathways, and transcriptional regulation that may be related to heart repair. Future studies are needed to understand how ECs interact with immune components during the reparative process after cardiac injury and during human HF. Further elucidating the role of ECs in regulating the immune response to cardiac injury in murine and human HF may provide novel insights into cellular mechanisms for alleviating both acute and chronic cardiac inflammation. This will not only provide a better understanding of the underlying biology of human heart disease, but it will pave the way for developing novel therapeutic targets.

## Materials and Methods

### Ethics approval for the use of donated tissues

Cardiac tissues and blood samples used in this study were collected during cardiothoracic surgeries performed at Texas Children’s Hospital (Houston, Texas). The protocols for the procurement and use of these patient samples were approved by the Institutional Review Board for Baylor College of Medicine and Affiliated Hospitals (Protocol Number H-26502). With the help of the Heart Center Biorepository at Texas Children’s Hospital, consent was obtained from patients with various forms of pediatric heart disease, including cardiomyopathies. The anatomic location of the tissue collected was based on the specific surgical repair being performed. This information, along with more specific patient information, can be found in Table S1.

### Sample collection and preservation

Cardiac tissue and blood samples were collected in the operating room during various pediatric cardiovascular surgeries. Cardiac tissue samples were kept in cold saline on ice during transfer to the laboratory for preservation. Before cardiac bypass was initiated, blood samples were collected into EDTA-coated vacutainers and were then transferred to the laboratory on ice. Cardiac tissue samples were carefully dissected into multiple aliquots, some of which were flash-frozen and stored at −80°C and others of which were fixed in 10% neutral buffered formalin for 16–24 h at 4°C. Formalin-fixed samples were serially dehydrated and embedded in paraffin blocks for histology. Formalin-fixed paraffin-embedded (FFPE) samples were then used to make a tissue microarray (2-mm cores) for high-throughput image analysis.

### Histology

Tissue sections were deparaffinized at 60°C for 1 h and then dewaxed and rehydrated in a graded series of alcohol. For hematoxylin and eosin (H&E) staining, tissue sections were incubated with hematoxylin for 15 min, followed by incubation with acidified Eosin Y for 3 min at RT. The samples were re-paraffinized, dried, and mounted before imaging. Masson’s Trichrome staining was performed according to manufacturer’s instructions (HT15; Sigma-Aldrich). Images were acquired using the Cytation 5 Cell Imaging Multi-Mode Reader (Biotek). Contrast of both H&E and Masson’s Trichrome images were enhanced using the auto-contrast function in Adobe Photoshop, with the same contrast settings applied to each image for consistency.

### IMC

The tissue microarray of FFPE cardiac samples was used for IMC analysis. Tissue sections were warmed at 60°C for 1 h and dewaxed in three separate xylene washes for 10 min each. The tissue sections were then rehydrated in a graded series of alcohol (ethanol:deionized water 100:0, 100:0, 96:4, 90:10, 80:20, 70:30) for 5 min each and then in PBS for 10 min. Epitope retrieval was performed in Tris EDTA retrieval buffer (pH 9; GeneMed) at 95°C for 20 min, after which, the slides were immediately cooled in TBS for 20 min. Samples were blocked in 3% BSA and 10% donkey serum in PBS with Tween (PBST) for 2 h at RT. Incubation with the antibody panel was performed in blocking buffer overnight at 4°C. Tissue samples were washed twice with TBST and twice with TBS. Slides were incubated with Intercalator-Ir solution for 5 min at RT and washed twice with TBS. The samples were dipped in water and dried before obtaining IMC measurements. The antibody panel targeted functional markers for DNA damage, immune regulation, cell cycle, and phenotypic markers to identify epithelial, endothelial, mesenchymal, and immune cell types. All conjugated antibodies used in this study can be found in Table S7. The Hyperion Imaging System (Fluidigm) was used for IMC image acquisition. The largest square area was selected from the center of each tissue microarray core for laser ablation. Commercial acquisition software (Fluidigm) was used to preprocess raw data and monitor acquisition quality.

### IMC data analysis

MCD Viewer was used to process and convert data to TIFF format. Images were further enhanced using the median noise and sharpen functions in Adobe Photoshop.

### ISG analysis

We directly downloaded publicly available human data that were previously processed and annotated (Wang et al, 2020) and performed standard data normalization and visualization using the Seurat software suite. Specifically, we computed 50 principal components (PCs) from the top 2,000 highly variable genes. We then performed batch effect correction on the PC dimension by using RunHarmony function and projected cells on the UMAP dimension for visualization by using RunUMAP.

The ISG analysis was based on previously reported ISGs (Schneider et al, 2014; Calcagno et al, 2020). A total of 96 ISGs were included for scoring. A detailed gene list is provided in Table S3. The Seurat “AddModuleScore” function was used to calculate ISG scores. This method scores the expression of ISGs by normalizing expression levels of ISGs against those of randomly selected background genes with similar expression levels. The size of the background gene pool is 10 times that of the input gene set. ISG scores that fit Gaussian distribution across cells were transformed into Z-scores for plotting and statistical significance testing. Differences of ISG scores and PD-L1 expression between control ECs and ECs from patients with DCM or congestive heart failure were tested for significance using the nonparametric Wilcoxon signed-rank test.

### Experimental animals

All murine experiments were performed under the Baylor College of Medicine Institutional Animal Care and Use Committee protocol number 5713. ICR mice were acquired from the Center for Comparative Medicine at Baylor College of Medicine and used for all surgical experiments. Female mice were set up for timed pregnancy to deliver pups for surgical procedures at postnatal day 1 or 8 (P denotes postnatal day, i.e., days after birth).

### LAD-O of neonatal mice

Neonatal mice were subjected to MI via LAD-O surgery as previously described (Porrello et al, 2011). Pups were subjected to hypothermic anesthetization before procedures. Feet pinching was used to evaluate proper anesthetization. Nylon sutures (8-0 nonabsorbable) were used for LAD occlusion. Proper occlusion was indicated by blanching of the affected myocardium. The thoracic cavity was closed with vicryl sutures (6-0 absorbable). Duration of the surgery from hypothermic induction to recovery was around 10 min. Sham procedures are identical in all aspects except for occlusion of the LAD. Mice were euthanized 4 d after surgery. Hearts were harvested and digested into single-cell suspensions to be used for single-cell RNA and single-nuclei ATAC sequencing.

### Isolation and enrichment of CD31^+^ single cells and nuclei from murine cardiac tissue

For scRNA-seq and snATAC-seq, the atria and aorta were removed from the heart, and the remaining tissue was used for single-cell digestion. Cells were harvested at either P5 or P12, from pools of five hearts subjected to MI at P1 or P8, respectively. Tissues were minced into small pieces in digestion buffer (1 mg/ml collagenase A; HBSS) and incubated at 37°C with agitation for 30 min. The suspension was triturated with a 5-ml pipette every 10 min. Proper digestion was verified by the appearance of single cells under a microscope. The digestion was then diluted 1:1 with HBSS, and the resulting cell suspension was centrifuged at 300*g* for 6 min to remove cardiomyocytes. Supernatant was passed through a 40-micron filter to remove tissue fragments and cardiomyocytes, and then centrifuged at 500*g* for 6 min. The cell pellet was resuspended in 1 ml of MACs buffer (PBS, 0.5% BSA, 2 mM EDTA). The resuspended cells were centrifuged at 500*g* for 6 min to remove residual buffer and resuspended in a final volume of 80 μl for incubation with 20 μl of CD31 MACs beads (Miltenyi Biotech). MACs enrichment for CD31^+^ cells was performed according to the Miltenyi enrichment protocol. Live cells were quantified with Trypan Blue and in Cyto C-Chip DHC-F01. For single-nuclei ATAC sequencing, nuclei were isolated from cells according to the 10x protocol. Live nuclei were quantified with DAPI and in Cyto C-Chip DHC-F01.

### Isolation of single cells and nuclei from murine cardiac tissue

Neonatal hearts were harvested and washed with ice-cold Tyrode’s solution. The left ventricle was removed and diced with curved scissors. The tissue was then digested with Collagenase A at 37°C until single-cell dissociation was achieved (∼20 min). Dissociated cells were diluted to a concentration of 200 cells per μl in PBS with 0.01% BSA. Drop-seq was then performed as previously described (Macosko et al, 2015). Cells were co-encapsulated into nano-liter–sized droplets containing barcoded microparticles (catalog number Macosko201110; ChemGenes) and lysis buffer using a custom microfluidics device (FlowJEM). After droplet breakage, reverse transcription (Thermo Fisher Scientific), and exonuclease treatment (NEB), all cDNA was PCR-amplified (KAPA), pooled, purified with Ampure XP beads (Beckman Coulter), and run on a fragment analyzer (Advanced Analytical Technologies, Inc.) for quality control, quantification, and size determination. Library preparation was performed with the Illumina Nextera XT kit, and libraries were triple-purified with Ampure XP beads (Beckman Coulter). All libraries were sequenced on an Illumina NextSeq500 instrument.

Nuclear isolation was performed as previously described, with the following specifications (Mo et al, 2015). Briefly, fresh cardiac tissue was harvested on ice and was immediately homogenized with a Biogen Series PRO200 (PRO Scientific) before performing Dounce homogenization in HB buffer (0.25 M sucrose, 25 mM KCl, 5 mM MgCl2, 20 mM Tricine-KOH, pH 7.8; with protease inhibitors; 1 mM DTT; 0.15 mM spermine; 0.5 mM spermidine, and RNAse inhibitors) with 5% IGEPAL CA-630. Nuclei were isolated via density gradient centrifugation with optiprep density gradient medium after mixing homogenate 1:1 with a 50 iodoxinal (5 vol Optiprep [Cat#D1556; Sigma-Aldrich] with 1 vol diluent [150 mM KCl; 30 mM MgCl_2_; 120 mM Tricine-KOH; pH 7.8]). After centrifugation at 10,000G for 18 min, all nuclei isolated from the 30–40% interface were recovered and washed before performing snATAC-seq.

### scRNA and snATAC sequencing

scRNA-seq libraries were generated using the 10x Chromium Single Cell 3′ Library Kit v3 (10x Genomics) according to the manufacturer’s instructions. We diluted cell suspensions for a target of 10,000 cells for each library. snATAC-seq libraries were generated using the 10x Chromium Single Cell ATAC Library Kit v1 according to the manufacturer’s instructions. Final quantification and quality control were determined by using Quibit and Fragment Analyzer. Nuclei suspensions were diluted for a target of 10,000 nuclei per library. Libraries were sequenced on an Illumina NextSeq 500 at the Baylor College of Medicine or on a HiSeq Xten Illumina platform (Novaseq Illumina platform) by Novogene.

### scRNA-sequencing analysis

The 10x Genomics Cell Ranger software (cellranger 3.0.1, www.10xgenomics.com) was used to handle raw sequencing data. Fastq files were aligned to the mouse mm10 genome. A pre-mRNA version of the reference genome was provided for gene count quantification with the Cellranger count function. CellBender v0.1.0 was used to remove background from the Cellranger output with the remove-background module in default settings. The h5 expression matrices from each data source were then imported into Seurat 3 for downstream processing and annotation to account for source-specific quality differences (Stuart et al, 2019). Single cells were filtered for UMI (unique molecular identifiers) counts with a minimum of 500 and a maximum of 25,000 per cell. We also imposed a cutoff of 0.5% for mitochondrial reads and a cutoff of 0.25% for doublet score. Seurat toolkit version v.4.0 in R v.3.8 was used to perform downstream analyses. The processed UMI count matrices of filtered cells were normalized with the Seurat function SCTransform to regress out additional variation.

The FindVariableFeatures function was used to calculate the top 5,000 most variable features for each sequenced library. The Seurat function FindIntegrationAnchors was used to find mutual nearest neighbors across subsets. Seurat’s IntegrateData function was used to construct an integrated matrix for each cell with a correction vector (based on anchor and similarity scores). The integrated data were scaled and the principal components were calculated (n = 50). Dimensional reduction (UMAP) and clustering were used to identify clusters (dimensions = 20, cluster resolution = 1.5).

For the whole heart dataset, 13,504 cells were generated across 4 experiments, with an average sequencing depth of 1,736 reads per cell, 1,031 genes per cell (Table S2). We identified cardiomyocytes based on expression of myofibrillar markers *Tnnt2* and *Myl6* and cardiac fibroblasts based on expression of fibroblast and extracellular matrix markers *Col1a1*, *Tcf21*, and *Dcn*. SMCs were distinguished by the expression of markers *Acta2*, *Eln*, and *Rgs5*, whereas pericytes were identified by *Pdgfrb* expression. Immune cells were identified by expression of *Lyz2* and *Cx3cr1* in macrophages, and *Cd52*, *Trbc2*, and *Cd3d* in T-cells. ECs were enriched for markers such as *Fabp4* and *Pecam-1*, whereas endocardial cells expressed *Nrp3* and *Igf2*. Epicardial cells were defined by the expression of *Saa3* and other unique markers.

For the CD31+-enriched dataset, we excluded contaminating nonendothelial cells, which were characterized by the expression of fibroblast (*Col1a1*, *Tcf21*), cardiomyocyte (*Tnnt2*, *Myh6*), red blood cell (*Hbb-bt*, *Hba-a1*, *Hbb-bs*), macrophage (*Lyz2*, *Cx3cr1*), T cell (*Cd52*, *Trbc2*, *Cd3d*), B cell (*Ighm*, *Igkc*), pericyte (*Pdgfrb*) or SMC (*Acta2*) markers. To further remove doublets, FindAllMarkers was used to identify clusters of cells that express more than 1 major cell type marker. We identified endocardial cells based on the distinct expression of *Npr3*, *Dcn*, and *Postn*, and lymphatic ECs based on the expression of canonical markers *Ccl21a*, *Prox1*, *Pdpn*, and *Fgl2* (Fig S3A and Table S5). ECs were identified by the expression of pan-endothelial markers, such as *Fabp4* and *Pecam-1*, which encodes CD31. Arterial ECs were positive for *Gja4*, *Gja5*, *Sox17*, and *Hey1* (Fig S3A, Table S5), whereas venous ECs expressed markers such as *Vwf*, *Nr2f2*, and *CouptfII* (Fig S3A, Table S5). Capillary venous ECs positive for *Egr1*, *Fos*, and *JunB* were also detected, although capillary ECs showed the least overall specificity in terms of unique gene expression and were broadly enriched for markers such as *Gpihbp1*, *Rgcc*, and *Car4* (Fig S3A, Table S5).

DEGs for each cell cluster were calculated with the Findmarkers function, Seurat implementation of the Wilcoxon rank-sum test (min.pct = 0.01, thresh.use = 0.25). Gene ontology analysis was performed with ShinyGO v0.75 (Ge et al, 2020). The CellChat v.1.1.2 R package was used to perform ligand–receptor analysis (Jin et al, 2021). Data were visualized by the *DoHeatmap* and *Dotplot* functions from Seurat.

### snATAC-sequencing analysis

The 10x Genomics Cell Ranger software (cellranger-atac-1.2.0) was used to handle raw sequencing data. Reads were mapped to the mouse mm10 genome. The peak outputs from each sample were aligned with cellranger-atac count and aggregated with cellranger-atac aggr (--nosecondary --normalize = none). MACS2 callpeak (v2.1.1.20160309) was used for initial clustering and per cluster and per sample peak calling (-f BAMPE -g mm -B -q 0.1). We then reaggregated samples with cellranger-atac aggr using a union set of peaks and imported them in Seurat for subsequent analysis. Low-quality nuclei with <15% of fragments (reads) in peaks were removed. Nuclei with a minimum of 30 K (few reads) and a maximum of 1,500 K (doublets/nuclei clumps) peak region fragments were also removed.

To identify clusters in the snATAC-seq data, we transferred cluster labels from scRNA-seq data. To accomplish this, we first identified common correlation patterns in the gene activity matrix, which is the summation of fragments intersecting with the gene body and promoter region, from both datasets. Mapping of fragments of each cell to the 2-kb upstream region of gene coordinates is performed with the GeneActivity function in Seurat. The FindTransferAnchors function was used to identify patterns between the two experimental conditions for integration. Dimensionality reduction was performed with Harmony to correct principal component analysis embeddings. Each snATAC-seq nuclei was assigned scores on the basis of cluster labels from the scRNA-seq data.

### Computing NMS

The motif scanning tool FIMO was used with motifs from the CisBP (v2.0.0) database to identify peaks with motif matches (Grant et al, 2011; Weirauch et al, 2014). We used the standard *P*-value cutoff of 1 × 10^−4^ for motif matching in FIMO (Maurano et al, 2012; Whyte et al, 2013; Kheradpour & Kellis, 2014). The Seurat::AverageExpression (slot = “counts”) function was used to calculate the average accessibility count within each population. For a given TF, a gene’s NMS (i.e., NMS) was computed as a weighted summation of the TF’s motif counts in the peaks assigned to the gene. In particular, the NMS for gene and TF was computed as follows: for the -th peak assigned to the gene, denotes the peak’s accessibility score, denotes the motif count of TF in the peak, and denotes the peak’s length in kilobases.

### TF–TF co-localization and network analysis

For the TFs having motif matches in the ATAC peaks, we obtained their families from the CisBP (v2.0.0) database. We then computed the Pearson’s correlation coefficient between every pair of families’ motif counts across the ATAC peaks and created a network where each node indicates a motif family, and the edges represent the Pearson’s correlation coefficient values between families. To identify the groups of colocalizing TF families, we then applied the igraph package’s spectral community detection algorithm on these networks (https://igraph.org/).

### Single-molecule fluorescence in situ hybridization with RNAscope probes

FFPE murine cardiac tissue samples were sectioned into 5-μm thick slides. The slides were stained using the RNAscope 2.5 HD Assay-RED protocol (ACD) according to the manufacturer’s instructions. Nuclei were counterstained for 10 min at RT with DAP. Endothelial cells were stained with Isolectin B4 (Vector Laboratories). The following probes were used for analysis: Probe-Mm-Iigp1 (Cat No. 520771-C2) and Probe-Mm-Stat1 (Cat No. 479611-C2). Slides were imaged with a Zeiss LSM880 confocal microscope. Visualization and image processing was performed with FIJI/ImageJ software.

### Study approval

All murine experiments were performed under the Baylor College of Medicine Institutional Animal Care and Use Committee protocol number 5713. Human cardiac tissue collection was approved by the Institutional Review Board for Baylor College of Medicine and Affiliated Hospitals (Protocol Number H-26502) and collection occurred during cardiothoracic surgeries performed at Texas Children’s Hospital.

## Data Availability

All sequencing data generated in this study will be deposited to the National Center for Biotechnology Information (NCBI) Gene Expression Omnibus (GEO; https://www.ncbi.nlm.nih.gov/geo/).

## Supplementary Material

Reviewer comments
